# Sequencing of *Treponema pallidum* subsp. *pallidum* from isolate UZ1974 using Anti-Treponemal Antibodies Enrichment: First complete whole genome sequence obtained directly from human clinical material

**DOI:** 10.1371/journal.pone.0202619

**Published:** 2018-08-21

**Authors:** Linda Grillová, Lorenzo Giacani, Lenka Mikalová, Michal Strouhal, Radim Strnadel, Christina Marra, Arturo Centurion-Lara, Lucy Poveda, Giancarlo Russo, Darina Čejková, Vladimír Vašků, Jan Oppelt, David Šmajs

**Affiliations:** 1 Department of Biology, Faculty of Medicine, Masaryk University, Brno, Czech Republic; 2 Department of Medicine, Division of Allergy and Infectious Diseases, University of Washington, Seattle, United States of America; 3 Department of Dermatovenerology, University Hospital Brno, Brno, Czech Republic; 4 Functional Genomics Center Zurich, University of Zurich, Zurich, Switzerland; 5 Department of Immunology, Veterinary Research Institute, Brno, Czech Republic; 6 1^st^ Dermatovenereological Clinic St. Anne´s University Hospital Brno, Faculty of Medicine, Masaryk University, Brno, Czech Republic; 7 CEITEC-Central European Institute of Technology, Masaryk University, Brno, Czech Republic; 8 National Centre for Biomolecular Research, Faculty of Science, Masaryk University, Brno, Czech Republic; National Institutes of Health, UNITED STATES

## Abstract

*Treponema pallidum* subsp. *pallidum* (TPA) is the infectious agent of syphilis, a disease that infects more than 5 million people annually. Since TPA is an uncultivable bacterium, most of the information on TPA genetics comes from genome sequencing and molecular typing studies. This study presents the first complete TPA genome (without sequencing gaps) of clinical isolate (UZ1974), which was obtained directly from clinical material, without multiplication in rabbits. Whole genome sequencing was performed using a newly developed Anti-Treponemal Antibody Enrichment technique combined with previously reported Pooled Segment Genome Sequencing. We identified the UW074B genome, isolated from a sample previously propagated in rabbits, to be the closest relative of the UZ1974 genome and calculated the TPA mutation rate as 2.8 x 10^−10^ per site per generation.

## Introduction

*Treponema pallidum* subsp. *pallidum* (TPA) is the infectious agent of syphilis, a globally distributed, multi-stage, sexually transmitted disease with an annual incidence of more than 5.6 million cases, including about 350,000 cases of congenital syphilis [1, 2]. Since TPA cannot be continuously cultivated under *in vitro* conditions, most of the information on TPA genetics comes from genome sequencing studies and molecular typing studies [3–5].

The first complete genome sequence of TPA was published in 1998 [6] and since then, several other TPA genomes have been fully sequenced and analyzed (n = 6) [7–12]. In all these cases, treponemal DNA was isolated from bacteria propagated in experimentally infected rabbits. The low number of completely sequenced TPA genomes reflects the limited number of available TPA strains propagated in rabbits as well as the limited number of TPA strains for which the treponemal DNA was purified in sufficient amounts for whole genome sequencing.

For many years, there have been attempts to obtain whole genome sequences of TPA directly from clinical samples, without treponemal replication in rabbits. These attempts were mainly motivated by the need to i) characterize TPA strains causing modern syphilis infections and ii) compare strains isolated directly from patients and strains propagated in rabbits to reveal any potential adaptation of TPA to the rabbit host. For years, whole genome sequencing of TPA from clinical material was hindered by the very low number of treponemes in clinical specimens and the massive contamination of human and other DNA that precluded efficient sequencing of TPA directly from clinical samples. There is approximately 10^4^-times less TPA DNA copies present in clinical samples isolated directly from patients (10^1^–10^4^ of TPA DNA copies per μl of sample) compared to samples propagated in rabbits [13].

This limitation was resolved with the introduction of the Pooled Segment Genome Sequencing (PSGS) approach [14–17], which allowed whole genome sequencing to be performed with a very small number of TPA DNA copies per sample (10^3^–10^4^ of TPA DNA copies per μl of sample). Briefly, this method is based on specific amplification of overlapping fragments of TPA DNA (average size of *Treponema pallidum* (TP) intervals = 10 kb), which together represent the whole genome. To overcome the mis-assembly of short reads generated by Next-Generation Sequencing (NGS), TP intervals were divided into four different pools that undergo NGS separately. However, since this technique is quite time-consuming, other techniques for culture-independent selective TPA DNA enrichments were developed. These techniques, introduced in 2016, were based on RNA baits or DNA microarray capture to selectively enrich TPA DNA directly from clinical samples. Since then, the number of sequenced TPA genomes has increased dramatically. In total, 43 draft TPA genomes with coverage greater than 80% have been determined [18, 19]. However, the TPA capture techniques failed to produce complete genome sequences since i) the baits are only available against known TPA sequences and ii) reference-guided or whole genome sequence *de novo* assemblies using relatively short sequencing reads produced by NGS platforms cannot cover treponemal paralogous regions and regions containing tandem repeats. Paralogous regions (including *tpr* genes), two copies of nearly identical RNA operons, and regions containing repetitive sequences, which represent approximately 2% of the length of TPA whole genomes, can not be determined using these approaches.

This study presents a new culture-independent method to sequence TPA directly from human clinical material. The method, designated Anti-Treponemal Antibody Enrichment (ATAE), is based on selective separation of TPA on the cellular level. In this work, ATAE is coupled with the previously developed PSGS approach. This study presents the first complete genome of TPA obtained directly from clinical material, without multiplication in rabbits.

## Materials and methods

### Clinical characteristics of the UZ1974 sample and ethics statement

The UZ1974 sample was collected on December 29^th^, 2014, from a male patient with a primary genital chancre (Department of Dermatovenerology, University Hospital Brno, Czech Republic). Treponemes from the sample were used for anti-treponemal antibody-based enrichment, DNA isolation, whole genome DNA amplification, and direct or PSGS whole genome sequencing. This study was approved by the Ethics Committee of the Faculty of Medicine, Masaryk University (no. 25/2014); the patient signed an informed consent.

### Anti-Treponemal Antibodies Enrichment (ATAE)

#### Enrichment of TPA cells

TPA cells in the swab extract (PBS) were concentrated in the sample using polyclonal antibodies conjugated with biotin (Pierce^™^ *Treponema pallidum* Antibody PA1-73103; ThermoFisher Scientific, Waltham, MA, USA). Antibodies were bound to streptavidin coated magnetic beads (Dynabeads, CELLLection^™^ Biotin Binder Kit, ThermoFisher Scientific, Waltham, MA, USA). TPA cells were separated in the following steps: i) biotin-streptavidin binding (260 μl of beads, 240 μl of PBS, and 2 μl of anti-treponemal antibodies were mixed together and incubated at 4 °C overnight using end-over-end rotation); ii) removal of unbound antibodies (the mixture was twice washed with 500 μl of PBS); iii) incubation with swab extracts for one hour at room temperature (200 μl); iv) washing with PBS, and v) DNA isolation (QIAamp DNA Blood mini-kit, Qiagen, Hilden, Germany).

#### Whole genome amplification (WGA)

WGA was carried out using Multiple Displacement Amplification with phi 29 polymerase (REPLI-g Single Cell Kit, Qiagen, Hilden, Germany). The WGA products were purified using QIAEX II beads according to the manufacturer’s recommendations (Qiagen, Hilden, Germany).

### Quantification of TPA DNA and molecular typing

A nested PCR amplification (NPCR), with outer and inner primers targeting a single copy of the conserved treponemal gene encoding DNA polymerase (TP0105—*pol*A), was used to quantify the number of copies of TPA DNA in the clinical sample as described previously [20]. This NPCR protocol was able to detect 1–10 molecules of TPA DNA [20]. Multi Locus Sequence Typing (MLST) was also performed. MLST determined the allelic profile with the three-letter code, where the first letter corresponds to the TP0136 allele, the second to the TP0548 allele, and the third to the TP0705 allele [21].

### Next-Generation Sequencing (NGS) and processing of sequencing data from enrichment

NGS was performed at the Functional Genomics Center Zurich, University of Zurich, Switzerland. Briefly, the NEB Next Ultra DNA Library Prep for Illumina (New England Biolabs, MA, USA) was used as described below. The samples were end-repaired, and adapters were ligated to the fragmented DNA samples. The samples were purified using Agencourt AMPure XP (Beckman Coulter Inc., Brea, CA, USA). Fragments containing adapters on both ends were selectively enriched with PCR. The quality and quantity of the resulting libraries were validated using Qubit^®^ (1.0) Fluorometer and the Tapestation (Agilent, Waldbronn, Germany). The libraries were normalised to 10 nM and pooled equimolarly in Tris-Cl, pH 8.5 with 0.1% Tween 20. The resulting pool was sequenced on the Nextseq500 (Illumina, Inc, California, USA).

Quality of the raw reads was checked using FastQC [22]. The reads were pre-processed with the Cutadapt [23] and Fastx-toolkit [24]. First, the whole set of obtained reads was mapped to the human genome reference (hg38) and the human-matching reads were removed. Subsequently, the remaining reads were mapped to the TPA reference genome (GenBank Acc. No. CP004011.1). The mappings were performed using BWA MEM [25]. Mapping was post-processed using Samtools [26], Picard [27], and GATK [28]. Paralogous regions, regions containing repetitions and low-quality mappings were omitted from these analyses (mapping quality; MAPQ < 10). The overall mapping quality was checked using Samtools [26], Samstat [29], and Qualimap [30]. Alignment-guided genome assembly (alignment consensus) was generated using Samtools [26].

### Pooled Segment Genome Sequencing (PSGS)

A WGA reaction diluted fifty times served as a template for TP intervals amplification during the PSGS phase as described previously [14, 31].

### Sequencing of TPI regions and processing of sequencing data from PSGS and *de novo* assembly

The amplified TP intervals (n = 279) of the UZ1974 sample were NGS sequenced using the Illumina platform (NextSeq 500) at CEITEC (Brno, Czech Republic). Prior to NGS, the amplified TP intervals were labeled with multiplex identifier adapters and sequenced as four different samples to separate paralogous regions (Nextera^™^ XT DNA Sample Preparation Kit, Illumina Inc., Madison, WI, USA). The sequencing reads were trimmed (Trimmomatic, 0.32) [32], low quality bases were removed with a sliding window having a length of 4 nt, with an average quality of at least Phred = 17. When shorter than 50 bp, the sequencing reads were omitted from the analyses. Reads were analyzed with respect to four distinct pools and were *de novo* assembled using SeqMan NGen v4.1.0 software (DNASTAR, Madison, WI, USA) as well as mapped to the TPA reference genome (GenBank Acc. No. CP004011.1).

### Annotation of UZ1974 genome and nucleotide sequence accession number

For gene annotation, Geneious software v5.6.5 [33] was used. The *tpr*K gene showed intrastrain variability and the corresponding nucleotides positions were denoted as “N” (coordinates: 975981–976013; 976114–976171; 976280–976336; 976402–976423; 976509–976534; 976656–976690; 977125–977156). The complete genome sequence of the UZ1974 sample was deposited in GenBank under the accession number CP028438. Raw data are available in SRA under the following accession number: SRP156463.

### Clinical characteristics and analyses of the UW074B sample

The UW074B sample was isolated from a syphilis-infected patient on July 1^st^, 2004, in Seattle, USA. The UW074B represented a human whole blood sample that was inoculated to rabbits and underwent two passages. New Zealand white rabbits were used for propagation of the UW074B strain and experimental infections. Animal care was provided in full accordance with the Guide for the Care and Use of Laboratory Animals and experimental procedures were conducted under protocol 2198.05 approved by the University of Washington Institutional Animal Care and Use Committee (IACUC).

### Extraction of UW074B DNA from rabbit tissue

Spirochetes were extracted in sterile saline from infected rabbit testicles and collected in 15 ml tubes. The suspensions were spun at 1,000 rpm for 10 minutes to remove rabbit tissue debris. The supernatant was transferred to microcentrifuge tubes and the bacteria was pelleted at 12,000 g for 30 min at 4 °C. Pellets were resuspended in 200 μl of lysis buffer (10 mM Tris pH 8.0, 0.1 M EDTA, 0.5% sodium dodecyl sulfate), and DNA extracted using a DNA Mini Kit (Qiagen Inc., Chatsworth, CA) according to the manufacturer’s instructions.

### NGS and bioinformatic analyses of UW074B sample

Extracted DNA was sequenced at Covance (Redmond, WA, USA) using the Illumina MiSeq platform. Quality of the raw reads was checked using FastQC [22]. The reads were pre-processed using Cutadapt [23]. First, the whole set of obtained reads was mapped using bbmap [34] to the rabbit genome reference (OryCun2.0) and the rabbit-matching reads were removed. Subsequently, the remaining reads were mapped to the TPA reference genome (GenBank Acc. No. CP004011.1). The mapping was performed using BWA MEM [25]. Mapping was post-processed using Samtools [26], Picard [27], and GATK [28]. Paralogous regions, regions containing repetitions, and low-quality mappings were omitted from these analyses (mapping quality/MAPQ < 10). Secondary, an improperly paired alignments were removed as well. The overall mapping quality was checked using Samtools [26] and Qualimap [30]. To avoid cross-mapping, several post-alignment filtering steps were added using Samtools [26], Picard [27], and NGSUtils/Bamutils [35]. The filtering kept only alignments with minimum 35 bp aligned length, a maximum of 5% mismatches of the mapped read length and/or a maximum of 5 mismatches, a maximum of 5% of soft-clipping, and 0% hard-clipping of the total read length, and a MAPQ ≥ 40. Alignment-guided genome assembly (alignment consensus) was generated using Samtools [26].

### Phylogenetic analyses

Maximum likelihood phylogenetic trees were generated using MEGA 6 with the Tamura Nei model and 1000 pseudorandom bootstrap replicates [36].

## Results

### Clinical characteristics, molecular typing, and number of TPA DNA copies in the UZ1974 sample

The primary chancre swab was taken from the genital region of a heterosexual patient (UZ1974) with primary syphilis who was infected by a sexual worker. The sample was collected at the Department of Dermatovenerology, University Hospital Brno, Czech Republic, in 2014. The swab extract was frozen in 10% glycerol at −80 °C. As revealed by molecular typing (MLST), the UZ1974 isolate belonged the SS14-like group of TPA strains (allelic profile 1.26.1) and contained an A2058G mutation in the 23S rRNA genes leading to macrolide resistance. The UZ1974 isolate was completely identical to the SS14 reference genome at the TP0136 locus (GenBank Acc. No. CP004011.1; allelic variant 1), contained three single nucleotide variants (SNVs) at the TP0548 locus (allelic variant 26) and two SNVs at TP0705 locus (allelic variant 1) compared to the SS14 reference genome (GenBank Acc. No. CP004011.1). The swab extract of the primary chancre was positive for dark-field microscopy suggesting that a relatively large number of treponemes were present in the sample. Prior to enrichment, we estimated 10^3^ TPA DNA copies/μl, using established nested PCR protocol.

### Anti-Treponemal Antibodies Enrichment (ATAE)

**Fig 1:**
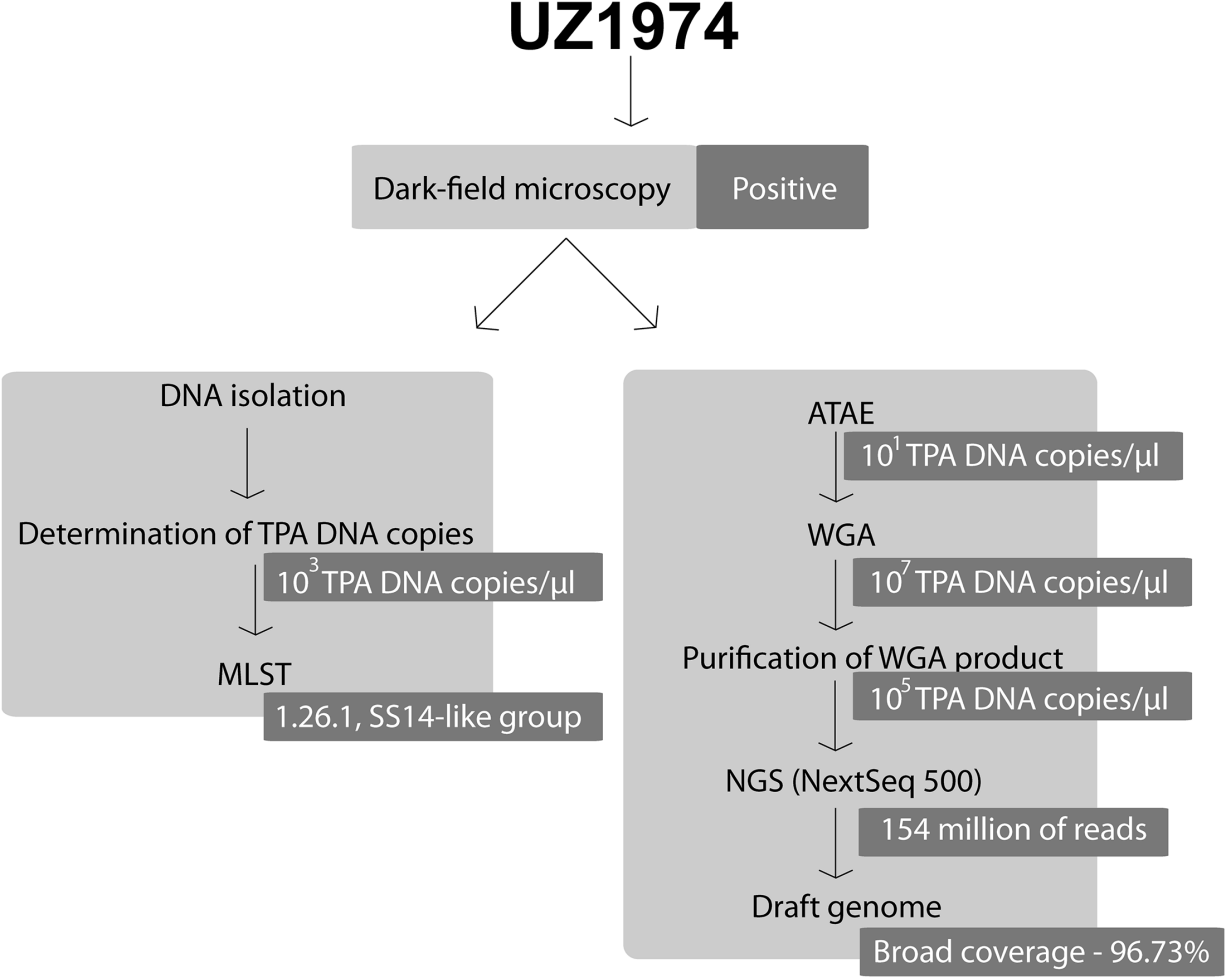
Workflow of ATAE coupled with PSGS. Dark-field microscopy, MLST, and determination of TPA DNA copies were performed on the UZ1974 clinical sample taken from a syphilis positive patient. TPA cells were concentrated in the sample using polyclonal antibodies conjugated with biotin, which were bound to streptavidin covered beads. Prior to NGS, whole genome amplification (WGA) was carried out using multiple displacement amplification using phi 29 polymerase; WGA products were purified using QIAEX II beads. The number of TPA DNA copies was monitored using the nested PCR protocol for *polA* detection [20]. Using the BWA MEM algorithm, the whole set of obtained reads from NGS (Illumina NextSeq 500) was mapped to the human genome reference (hg38), removed, and the rest of the reads were mapped to the TPA reference genome (GenBank Acc. No. CP004011.1).

The TPA cells present in the UZ1974 clinical sample were concentrated using polyclonal antibodies conjugated with biotin and bound to streptavidin coated magnetic beads (see Material and methods section). Following TPA enrichment, the total DNA was amplified with random primers and phi 29 polymerase (whole genome amplification; WGA). The WGA DNA products were then purified and sequenced using the Illumina platform (NextSeq 500). The workflow of ATAE and the whole DNA processing of clinical sample UZ1974 is shown in Fig 1.

As revealed by the pilot experiments done during ATAE development, the number of TPA DNA copies synthesized during WGA was directly dependent on the presence and concentration of contaminating (mostly human) DNA. Testing of WGA efficiency revealed that only a small amount of human DNA (3 ng) mixed with the positive control of TPA DNA (10 ng) decreased TPA amplification over 100 times (Grillová L., unpublished data). Moreover, in an unenriched UZ1974 clinical sample, the WGA procedure increased the number of TPA DNA copies by 2 orders of magnitude (to 10^5^ TPA DNA copies/μl). The UZ1974 sample enriched by ATAE revealed 10^1^ TPA DNA copies/μl before WGA and 10^7^ DNA copies/μl after WGA. WGA therefore increased the number of copies by 6 orders of magnitude. After DNA purification of WGA products, we were able to prepare an enriched UZ1974 sample with a total TPA DNA amount of 0.1 ng/μl relative to a total DNA concentration of 180 ng/μl., i.e. the sample contained 1,800 times more contaminating DNA than TPA DNA.

A total of 154 million Illumina reads were obtained. Since the UZ1974 isolate was in the SS14-like group of TPA strains, the genome sequence of the SS14 strain (GenBank Acc. No. CP004011.1) was used as the reference sequence during the reference-guided approach. A total of 198,765 reads mapped to the TPA SS14 reference corresponded to an average genome coverage depth of 24.76x. Broad coverage for UZ1974 was 96.73%. Other statistical data are presented in Table 1.

**Table 1:** NGS statistics for the UZ1974 genome obtained using ATAES.

NGS parameter	UZ1974
Total number of obtained reads after quality control	154 million
Reads mapped to the TPA SS14 genome reference (GenBank Acc. No. CP004011.1)	198,765
Average coverage depth	24.76x
Median coverage depth	9.55x
Broad coverage of TPA reference genome^a^	96.73%
Mean length of reads; range (bp)	142.54; 70–150

^a^The broad coverage calculated from positions with coverage ≥ 3.

#### PSGS

In parallel, the genome of UZ1974 was amplified using PSGS, which was used to verify the ATAE sequencing results. Moreover, PSGS unequivocally determined the chromosomal paralogous regions and regions containing repetitive sequences. The average sequencing coverage depth for all TP intervals (n = 279) was 1070.31x. Given that only 3.27% of the genome length was uncovered by ATAE in the UZ1974 genome, only 37 kbp had to be sequenced from the amplified intervals (TPI; n = 16) to obtain a complete genome sequence.

### Analysis of UZ1974 genome sequence

The TPA UZ1974 genome was found to be closely related to the TPA SS14 genome. The TPA UZ1974 genome contained fourteen 60 bp-long repetitions in the TP0433 gene (i.e., *arp*; acidic repeat protein), which is the same number found in the TPA SS14 *arp* gene. In addition, similarly to SS14, the UZ1974 genome showed the same structure of RNA operons, i.e., the sequence of 16S-5S-23S rRNA genes were identical in both operons and both had the same order of spacer pattern encoding tRNA-Ile / tRNA-Ala [37], within the first and second *rrn* operon, respectively. The 23S rDNA sequence in both operons harbored the A2058G mutation encoding resistance to macrolide antibiotics. In contrast to the SS14 genome containing ten 24 bp-long repetitions in the TP0470 gene (coding for a tetratricopeptide repeat containing protein) [38], there were eight 24 bp-long repetitions in the UZ1974 genome.

Compared to the TPA SS14 genome (GenBank Acc. No. CP004011.1), the UZ1974 genome differed in 18 single nucleotide variants (SNVs); 17 of which were found in genes (or in annotated open reading frames) and one was found in the intergenic region (Table 2). All but one of the SNVs located in open reading frames resulted in amino acid replacements in the corresponding proteins (Table 2). The majority of amino acid replacements were found in genes predicted to code for virulence factors, outer membrane proteins, and metabolic functions. In addition to SNV differences, there were 16 length differences in homopolymeric tracts between the SS14 and UZ1974 genomes (S1 Table).

**Table 2:** Identified SNVs between UZ1974 and SS14 genomes.

SNV^a^	ORF (Gene)	Gene product	Functional category	Syn/Nonsyn^b^
A94901C	TPASS_20085	PTS family fructose porter component IIA	Transport	Syn
G135108C	TPASS_20117	Tpr protein C	Virulence	Nonsyn (P534A)
C174177T	TPASS_20151	putative NADH dehydrogenase (ubiquinone), subunit RnfD	General metabolism	Nonsyn (V260I)
T333559C	IGR^c^	NA^d^	NA^d^	NA^d^
G342703A	TPASS_20324	putative outer membrane protein	Unknown	Nonsyn (A540T)
T364888C	TPASS_20341	UDP-N-acetylmuramate—L-alanine ligase	General metabolism	Nonsyn (L64P)
A522907G	TPASS_20488	methyl-accepting chemotaxis protein	Cell processes	Nonsyn (D195G)
C556154T	TPASS_0515	putative outer membrane protein	Unknown	Nonsyn (R456C)
G593294A	TPASS_20548	putative outer membrane protein	Unknown	Nonsyn (G53R)
G593298A	TPASS_20548	putative outer membrane protein	Unknown	Nonsyn (G53E)
A593912G	TPASS_20548	putative outer membrane protein	Unknown	Nonsyn (K145E)
C674219T	TPASS_20620	Tpr protein I	Virulence	Nonsyn (H46R)
A674227C	TPASS_20620	Tpr protein I	Virulence	Nonsyn (V48G)
T674233C	TPASS_20620	Tpr protein I	Virulence	Nonsyn (E51K)
T760092C	TPASS_20691	segregation and condensation protein ScpA	Cell processes	Nonsyn (K30R)
C772846T	TPASS_20705	bifunctional membrane carboxypeptidase/penicillin-binding protein	General metabolism	Nonsyn (M625V)
T773095C	TPASS_20705	bifunctional membrane carboxypeptidase/penicillin-binding protein	General metabolism	Nonsyn (G708S)
T861444C	TPASS_20793	hypothetical protein	Unknown	Nonsyn (L311R)

^a^Coordinates according to the SS14 reference genome (GenBank Acc. No. CP004011.1).

^b^Synonymous/nonsynonymous amino acid replacement.

^c^IGR—Intergenic region.

^d^NA—Not applicable.

### Comparison of the UZ1974 genome with the UW074B genomic sequence

A phylogenetic analysis of the whole genome sequence of the UZ1974 isolate (1,139,510 nt in length), with all available genome sequences from reference TPA strains and clinical isolates (n = 69; S2 Table, S1 and S2 Figs) [8–12, 18, 19, 39–41], revealed that UZ1974 and the draft genome sequence of TPA strain UW074B were closely related. To fully assess the genetic relatedness of UW074B, the genome was reassembled from the SRA data in the same way as the UZ1974 genome; the assembly covered 99.2% of the reference genome length (8,885 nt were not determined due to the paralogous character of the sequenced regions and/or due to the presence of repetitive sequences (S3 Table). A comparison of the complete genome sequences of both UZ1974 and UW074B revealed genetic difference at only one nucleotide position within the TP0548 gene (G vs. A; position 593,912 according to the TPA SS14 genome; CP004011.1). An additional genetic difference between the UZ1974 and UW074B genome sequences involved a 9-nt long repetition (TCCTCCCCC) in the TP0967 gene (between coordinates 1,051,840–1051,866; according to the TPA SS14 genome; CP004011.1). While the UZ1974 genome contained three such repetitions, the UW074B genome contained four. In addition, there were ten differences in the length of the homopolymeric tracts (S4 Table).

### TPA mutation rate derived from comparison of the UZ1974 and UW074B genomes

A single nucleotide difference detected in both analyzed genomes collected with 10.45 years between sample collection dates combined with the analyzed genome regions having a total length of 1,130,625 nt, corresponds to a mutation rate of 8.46 x 10^−8^ per nucleotide site per year. Since sites with intrastrain heterogeneity do not represent fixed mutations, they were excluded from the estimation of the TPA mutation rate. Similarly, expansions or reductions in the number of repetitive sequence motifs were not considered as mutations. Considering the long doubling time of TPA, equal to about 30 hours [42, 43], one can assume that 91,584 hours (10.45 years) between the isolation of the two samples corresponded to 2,748 treponemal generations. Assuming 292 generations per year, the estimated mutation rate number corresponds to a TPA mutation rate of 2.8 x 10^−10^ per site per generation.

## Discussion

Although new techniques allowing culture-independent selective TPA DNA enrichment [18, 19] coupled with NGS were developed in 2016, these techniques have fundamental limitations since they are based on RNA or DNA baits derived from previous sequencing data and could therefore enrich only DNA that is complementary to the DNA sequences used in the microarray or bead capture. Any potentially novel treponemal sequences will remain undetected during DNA enrichment. Therefore, our intention was to develop a technique that would not show such a bias. One possible solution for this problem is to directly sequence all the DNA from the sample, however, contamination with human DNA and other microbial DNA precludes efficient sequencing of treponemal DNA and complicates genome assembly in the chromosomal regions conserved among microbial species. In this study, we developed an Anti-Treponemal Antibodies Enrichment (ATAE) method based on enrichment of TPA cells using polyclonal anti-treponemal antibodies. The number of TPA and human DNA copies before and after whole genome amplification showed, that the enrichment step of TPA is quite efficient even though significant amounts of TPA DNA was lost during this step.

We used ATAE on one clinical sample taken from a syphilis positive patient. The TPA UZ1974 isolate (allelic profile 1.26.1), belonged to the SS14-like group of TPA [21, 44, 45] and represents one the most frequent allelic profiles found in the Czech Republic in recent years [46]. As with the SS14 strain, the TPA UZ4974 isolate harbored an A2058G mutation in both 23S rRNA genes resulting in resistance to macrolide antibiotics. According to Enhanced CDC-typing [47], UZ1974 was subtype “g” according to TP0548, representing the most frequent subtype found in Australia [48] and Europe (including the Czech Republic [46], Italy [49], Denmark [50], France [51], Ireland [47], and Switzerland [21]. At the same time, subtype “g” also belongs to the SS14-Ω (omega-cluster of SS14-like strains), which is currently spreading [18].

Despite ATAE being a useful technique, the enrichment was not as efficient as expected. Even though we tried several modifications of ATAE, we were unable to achieve a better treponemal to human cell ratio. Modifications to the ATAE protocol included i) different incubation times, ranging from 30 min to 1.5 hours at room temperature, ii) using monoclonal antibodies instead of polyclonal antibodies, iii) using a sepharose medium instead of magnetic beads, and iv) different numbers of washing steps. The ratio of TPA DNA to human DNA (HDNA) was about 1:1,800, which roughly corresponded to the human to TPA cell ratio, indicating that ATAE enriched the treponemal DNA 10-times compared to unenriched sample. As stated above, most of the total obtained reads belonged to the human genome and were excluded. The 43% of the remaining reads belonged to other bacteria (i.e., not to TPA), mostly to bacteria from the family *Prevotellaceae* that include bacteria isolated from many types of human material. When comparing ATAE enrichment efficiency to other available TPA culture-independent enrichments, including hybridization capture [18] and in-solution capture [19], ATAE had a similar or lower enrichment efficiency. Pinto and colleagues [19] were able to achieve a TPA/HDNA enrichment ratio of 1/1–1/100 while Arora and colleagues [18] were able to reach a TPA/HDNA enrichment ratio of 1/10-1/1000. Another ATAE disadvantage is linked to the fact that TPA cells need to be intact during the enrichment step, therefore, clinical samples need to be processed shortly after sampling (hours after sampling). On the other hand, when using ATAE, there is no introduction of a DNA enrichment bias as a consequence of a sequence-specific enrichment protocol. Moreover, enrichment on the cellular level has the potential to be used for transcriptomic and proteomic studies.

Irrespective of the culture-independent enrichments method used, including hybridization capture, in-solution capture, and ATAE, there is another problem with paralogous genome regions and regions containing repetitions precluding finishing of complete genome sequences. Many TPA genomes determined in our lab were sequenced by the PSGS technique [14–17] based on sequencing of amplified overlapping fragments covering the entire TPA genome. This method is quite laborious and time-consuming, however, until now, the only method, which is able to overcome the mis-assembly of short reads generated by NGS and thus truly determine the paralogous regions. In this study, we combined this approach with newly developed ATAE technique. The ATAE was able to generate only draft genome. The missing regions and paralogous regions were in the end established (the gaps were filled) with the sequencing data generated by PSGS. This allowed us to obtain the first complete genome sequence isolated directly from human material.

A phylogenetic analysis of the UZ1974 whole genome sequence, with all available genome sequences from reference TPA strains and clinical isolates (n = 69; S2 Table, S1 and S2 Figs), revealed that the UZ1974 and the TPA strain UW074B draft genome sequence were closely related. The mutation rate calculated from the UZ1974 and UW074B genomes corresponded to a TPA mutation rate of 2.8 x 10^−10^ per site per generation (assuming 292 generations per year), a number that is even lower that the recently estimated upper limit for the TPE mutation rate, i.e., 4.1 x 10^−10^ per site per generation [31]. In our previous work on yaws treponemes isolated from Ghana, Africa (TPE strain Ghana-051 and TPE CDC 2575, isolated 7.25 years apart), we estimated an upper mutation rate limit of 1.21 x 10^−7^ per nucleotide site per year (genome size: 1,139,577 nt). Since both strains, TPE Ghana-051 and TPE CDC 2575, had the same consensus genome sequence, the upper limit of the mutation rate in yaws treponemes was estimated as 4.1 x 10^−10^ per site per generation [31]. In this study, the mutation rate estimation assumes that the TPA present in the UW074B sample was directly transmitted to other patients that led to infection of patient UZ1974. In reality, this is not the most probable scenario. Instead, it is more likely that both patients were infected by descendants of a common ancestor of the UW074B and UZ1974 strains and the evolutionary distance between treponemes in both samples was therefore longer than the one used for mutation rate estimation. It is therefore likely that the real mutation rate is even lower, making this estimation of TPA mutation rate (2.8 x 10^−10^ per site per generation) probably close to the highest rate possible.

## Supporting information

S1 Fig Phylogeny for all TPA genome sequences available in GenBank. Maximum likelihood phylogenetic tree generated in MEGA 6 for genome-wide variable positions (n = 419) after excluding sites with missing data using all available TPA genomes (n = 69) and the examined UZ1974 genome. Draft genomes used had a broad coverage of 90% or more. Repetitive and paralogous regions were not included in the analyses.
(TIF)

S2 Fig Phylogeny for TPA genome sequences available in GenBank. Maximum likelihood phylogenetic tree generated in MEGA 6 for genome-wide variable positions (n = 1081) after excluding sites with missing data using available TPA genomes (n = 49) and the examined UZ1974 genome. Only draft genomes with broad coverage of 98.5% or more were used. Repetitive and paralogous regions were not included in the analyses.
(TIF)

S1 Table Differences in homopolymers found when comparing the UZ1974 isolate to the SS14 strain (GenBank Acc. No. CP004011.1). (DOCX)

S2 Table All available TPA genome sequences available in GenBank database. This data was used for phylogenetic tree reconstructions in S1 and S2 Figs.
(DOCX)

S3 Table A list of chromosomal regions from the UW074B genome sequence that were excluded from further analyses due to unambiguous mapping of the sequencing reads. Altogether, 8,885 nt out of the total genome length (0.8%) were not analyzed in the UW074B genome. In contrast to assembly of the UW074B genome, assembly of the UZ1974 genome sequence was based on PSGS, which allowed assembly of a complete genome sequence.
(DOCX)

S4 Table Differences in homopolymers found when comparing the UZ1974 isolate to the UW074B genome sequence. (DOCX)
