# Supplementary figures and images for: Sequencing of Treponema pallidum subsp. pallidum from isolate UZ1974 using Anti-Treponemal Antibodies Enrichment: First complete whole genome sequence obtained directly from human clinical material
Source: PLoS One. 2018 Aug 21;13(8):e0202619. doi: 10.1371/journal.pone.0202619 (PMC6103504; doi:10.1371/journal.pone.0202619)

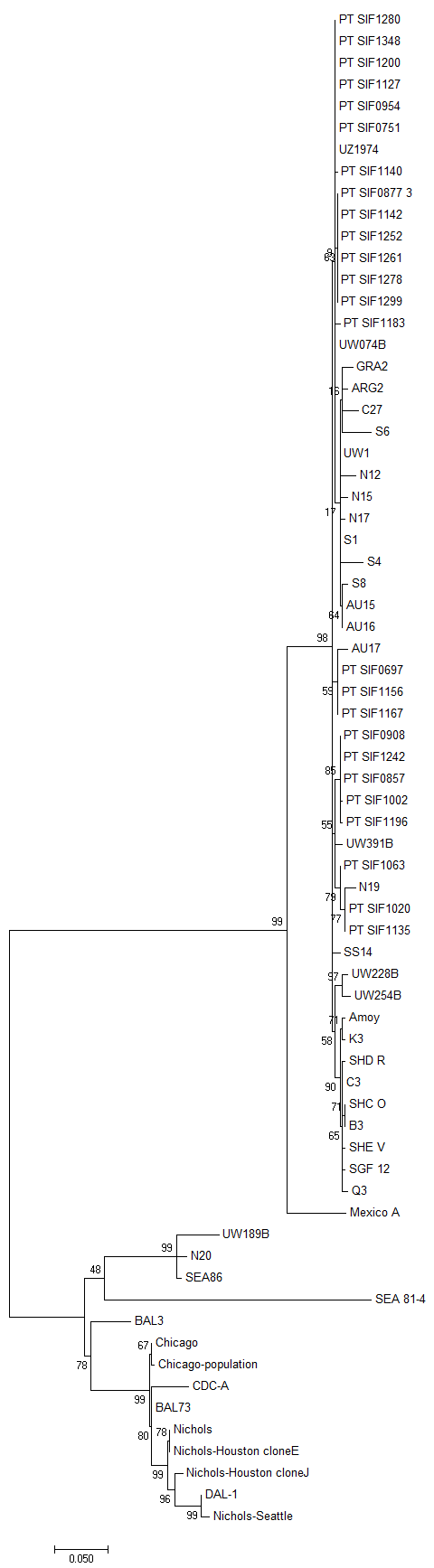

Supplement: S1 Fig — Maximum likelihood phylogenetic tree generated in MEGA 6 for genome-wide variable positions (n = 419) after excluding sites with missing data using all available TPA genomes (n = 69) and the examined UZ1974 genome. Draft genomes used had a broad coverage of 90% or more. Repetitive and paralogous regions were not included in the analyses. (TIF) [file pone.0202619.s001.tif]

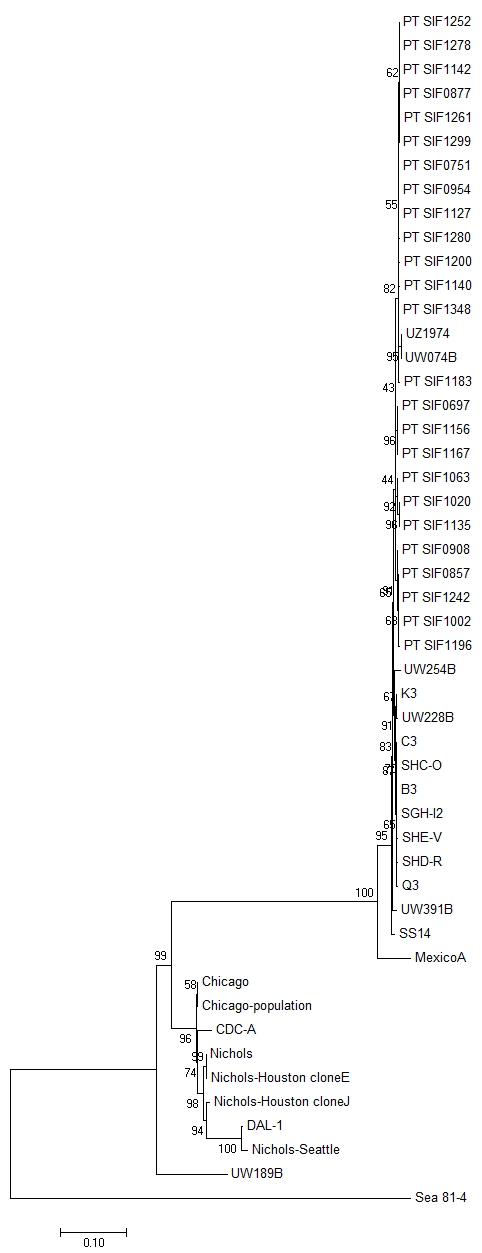

Supplement: S2 Fig — Maximum likelihood phylogenetic tree generated in MEGA 6 for genome-wide variable positions (n = 1081) after excluding sites with missing data using available TPA genomes (n = 49) and the examined UZ1974 genome. Only draft genomes with broad coverage of 98.5% or more were used. Repetitive and paralogous regions were not included in the analyses. (TIF) [file pone.0202619.s002.tif]
