# Supplementary material for: Sequencing of Treponema pallidum subsp. pallidum from isolate UZ1974 using Anti-Treponemal Antibodies Enrichment: First complete whole genome sequence obtained directly from human clinical material
Source: PLoS One. 2018 Aug 21;13(8):e0202619. doi: 10.1371/journal.pone.0202619 (PMC6103504; doi:10.1371/journal.pone.0202619)
Supplement: S2 Table — This data was used for phylogenetic tree reconstructions in S1 and S2 Figs. (DOCX) [file pone.0202619.s004.docx]

**S2 Table**. **All available TPA genome sequences available in GenBank database**. This data was used for phylogenetic tree reconstructions in Figs S1 and S2.

| Sample Name | Genetic group | Location | Year of isolation | Source | Genome (IDs) | References |
| --- | --- | --- | --- | --- | --- | --- |
| Nichols | Nichols-clade | USA | 1912 | rabbit inoculation | Complete (CP004010.2) | [11] |
| SS14 | SS14-clade | USA | 1977 | rabbit inoculation | Complete (CP004011.1) | [11] |
| Chicago | Nichols-clade | USA | 1951 | rabbit inoculation | Complete (CP001752.1) | [8] |
| Mexico A | SS14-clade | Mexico | 1953 | rabbit inoculation | Complete (CP003064.1) | [9] |
| DAL-1 | Nichols-clade | USA | 1991 | rabbit inoculation | Complete (CP003115.1) | [10] |
| SEA81-4 | Nichols-clade | USA | 1981 | rabbit inoculation | Complete (CP003679.1) | [12] |
| Amoy | SS14-clade | China | 2011 | rabbit inoculation | Draft^a^ | [39] |
| ARG2 | SS14-clade | Argentina | 2013 | clinical acquired | Draft^a^ | [18] |
| AU15 | SS14-clade | Austria | 2013 | clinical acquired | Draft^a^ | [18] |
| AU16 | SS14-clade | Austria | 2013 | clinical acquired | Draft^a^ | [18] |
| AU17 | SS14-clade | Austria | 2013 | clinical acquired | Draft^a^ | [18] |
| CZ27 | SS14-clade | Czech Republic | 2012 | clinical acquired | Draft^a^ | [18] |
| GRA2 (Grady) | SS14-clade | USA | 1980-1999 | rabbit inoculation | Draft^a^ | [18] |
| NE12 | SS14-clade | The Netherlands | 2013 | clinical acquired | Draft^a^ | [18] |
| NE15 | SS14-clade | The Netherlands | 2013 | clinical acquired | Draft^a^ | [18] |
| NE17 | SS14-clade | The Netherlands | 2013 | clinical acquired | Draft^a^ | [18] |
| NE19 | SS14-clade | The Netherlands | 2013 | clinical acquired | Draft^a^ | [18] |
| SW1 | SS14-clade | Switzerland | 2012 | clinical acquired | Draft^a^ | [18] |
| SW4 | SS14-clade | Switzerland | 2012 | clinical acquired | Draft^a^ | [18] |
| SW6 | SS14-clade | Switzerland | 2012 | clinical acquired | Draft^a^ | [18] |
| SW8 | SS14-clade | Switzerland | 2012 | clinical acquired | Draft^a^ | [18] |
| UW249B | SS14-clade | USA | 2004 | rabbit inoculation | Draft^a^ | [18] |
| BAL3 | Nichols-clade | USA | 1973 | rabbit inoculation | Draft^a^ | [18] |
| BAL73 | Nichols-clade | USA | 1973 | rabbit inoculation | Draft^a^ | [18] |
| NE20 | Nichols-clade | Netherlands | 2013 | clinical acquired | Draft^a^ | [18] |
| SEA86-1 | Nichols-clade | USA | 1986 | rabbit inoculation | Draft^a^ | [18] |
| SHC_O | SS14-clade | China | 2014 | rabbit inoculation | Draft^a^ | [40, 41] |
| SHD_R | SS14-clade | China | 2014 | rabbit inoculation | Draft^a^ | [40, 41] |
| SHE_V | SS14-clade | China | 2014 | rabbit inoculation | Draft^a^ | [40, 41] |
| SGF_12 | SS14-clade | China | 2014 | rabbit inoculation | Draft^a^ | [40, 41] |
| B3 | SS14-clade | China | 2015 | rabbit inoculation | Draft^a^ | [40, 41] |
| C3 | SS14-clade | China | 2015 | rabbit inoculation | Draft^a^ | [40, 41] |
| K3 | SS14-clade | China | 2015 | rabbit inoculation | Draft^a^ | [40, 41] |
| Q3 | SS14-clade | China | 2015 | rabbit inoculation | Draft^a^ | [40, 41] |
| PT_SIF1348 | SS14-clade | Portugal | 2014 | clinical acquired | Draft^a^ | [19] |
| PT_SIF1127 | SS14-clade | Portugal | 2013 | clinical acquired | Draft^a^ | [19] |
| PT_SIF1135 | SS14-clade | Portugal | 2013 | clinical acquired | Draft^a^ | [19] |
| PT_SIF1140 | SS14-clade | Portugal | 2013 | clinical acquired | Draft^a^ | [19] |
| PT_SIF1142 | SS14-clade | Portugal | 2013 | clinical acquired | Draft^a^ | [19] |
| PT_SIF1156 | SS14-clade | Portugal | 2013 | clinical acquired | Draft^a^ | [19] |
| PT_SIF1167 | SS14-clade | Portugal | 2013 | clinical acquired | Draft^a^ | [19] |
| PT_SIF1183 | SS14-clade | Portugal | 2013 | clinical acquired | Draft^a^ | [19] |
| PT_SIF1196 | SS14-clade | Portugal | 2013 | clinical acquired | Draft^a^ | [19] |
| PT_SIF1200 | SS14-clade | Portugal | 2013 | clinical acquired | Draft^a^ | [19] |
| PT_SIF1242 | SS14-clade | Portugal | 2014 | clinical acquired | Draft^a^ | [19] |
| PT_SIF1299 | SS14-clade | Portugal | 2014 | clinical acquired | Draft^a^ | [19] |
| PT_SIF1252 | SS14-clade | Portugal | 2014 | clinical acquired | Draft^a^ | [19] |
| PT_SIF1261 | SS14-clade | Portugal | 2014 | clinical acquired | Draft^a^ | [19] |
| PT_SIF1278 | SS14-clade | Portugal | 2014 | clinical acquired | Draft^a^ | [19] |
| PT_SIF1280 | SS14-clade | Portugal | 2014 | clinical acquired | Draft^a^ | [19] |
| PT_SIF0877_3 | SS14-clade | Portugal | 2010 | clinical acquired | Draft^a^ | [19] |
| RP_SIF0751 | SS14-clade | Portugal | 2009 | clinical acquired | Draft^a^ | [19] |
| PT_SIF0857 | SS14-clade | Portugal | 2010 | clinical acquired | Draft^a^ | [19] |
| PT_SIF0697 | SS14-clade | Portugal | 2009 | clinical acquired | Draft^a^ | [19] |
| PT_SIF0908 | SS14-clade | Portugal | 2010 | clinical acquired | Draft^a^ | [19] |
| PT_SIF0954 | SS14-clade | Portugal | 2010 | clinical acquired | Draft^a^ | [19] |
| PT_SIF1002 | SS14-clade | Portugal | 2011 | clinical acquired | Draft^a^ | [19] |
| PT_SIF1020 | SS14-clade | Portugal | 2011 | clinical acquired | Draft^a^ | [19] |
| PT_SIF1063 | SS14-clade | Portugal | 2013 | clinical acquired | Draft^a^ | [19] |
| Chicago-population | Nichols-clade | USA | 1951 | rabbit inoculation | Draft^a^ | Unpublished data, available in GenBank |
| CDC-A | Nichols-clade | USA | 2013 | rabbit inoculation | Draft^a^ | Unpublished data, available in GenBank |
| Nichols-Seattle | Nichols-clade | USA | 1912 | rabbit inoculation | Draft^a^ | Unpublished data, available in GenBank |
| Nichols-Houston_cloneE | Nichols-clade | USA | 2015 | rabbit inoculation | Draft^a^ | Unpublished data, available in GenBank |
| Nichols-Houston_cloneJ | Nichols-clade | USA | 2015 | rabbit inoculation | Draft^a^ | Unpublished data, available in GenBank |
| UW074B | SS14-clade | USA | 2004 | rabbit inoculation | Draft^a^ | Unpublished data, available in GenBank |
| UW189B | Nichols-clade | USA | 2004 | rabbit inoculation | Draft^a^ | Unpublished data, available in GenBank |
| UW228B | SS14-clade | USA | 2004 | rabbit inoculation | Draft^a^ | Unpublished data, available in GenBank |
| UW254B | SS14-clade | USA | 2004 | rabbit inoculation | Draft^a^ | Unpublished data, available in GenBank |
| UW391B | SS14-clade | USA | 2006 | rabbit inoculation | Draft^a^ | Unpublished data, available in GenBank |

^a^Genome broad coverage was at least 90%, with at least 3 reads per covered site was required.
