# Supplementary material for: Sequencing of Treponema pallidum subsp. pallidum from isolate UZ1974 using Anti-Treponemal Antibodies Enrichment: First complete whole genome sequence obtained directly from human clinical material
Source: PLoS One. 2018 Aug 21;13(8):e0202619. doi: 10.1371/journal.pone.0202619 (PMC6103504; doi:10.1371/journal.pone.0202619)
Supplement: S3 Table — Altogether, 8,885 nt out of the total genome length (0.8%) were not analyzed in the UW074B genome. In contrast to assembly of the UW074B genome, assembly of the UZ1974 genome sequence was based on PSGS, which allowed assembly of a complete genome sequence. (DOCX) [file pone.0202619.s005.docx]

**S3 Table. A list of chromosomal regions from the UW074B genome sequence that were excluded from further analyses due to unambiguous mapping of the sequencing reads.** Altogether, 8,885 nt out of the total genome length (0.8%) were not analyzed in the UW074B genome. In contrast to assembly of the UW074B genome, assembly of the UZ1974 genome sequence was based on PSGS, which allowed assembly of a complete genome sequence.

| Gene | Coordinates in the TPA SS14 genome; CP004011.1 | Length of excluded region (nt) |
| --- | --- | --- |
| *tpr*C | 135,942-136,678 | 737 |
| *rrn*1 interspacer | 232,961-233,024 | 64 |
| *rrn*2 interspacer | 232,961-233,024 | 74 |
| *tpr*E | 330,936-331,393 | 458 |
| TP0315 | 331,600-332,385 | 786 |
| *tpr*F | 332,535-334,241 | 1,708 |
| *tpr*G | 334,963-335,829 | 867 |
| *arp* | 462,274-463,121 | 848 |
| TP0470 | 498,879-499,134 | 256 |
| *tpr*I | 673,604-674,340 | 737 |
| *tpr*J | 675,905-676,673 | 769 |
| *tpr*K | 975,797-977,377 | 1,581 |
