# Supplementary material for: Sequencing of Treponema pallidum subsp. pallidum from isolate UZ1974 using Anti-Treponemal Antibodies Enrichment: First complete whole genome sequence obtained directly from human clinical material
Source: PLoS One. 2018 Aug 21;13(8):e0202619. doi: 10.1371/journal.pone.0202619 (PMC6103504; doi:10.1371/journal.pone.0202619)
Supplement: S4 Table — (DOCX) [file pone.0202619.s006.docx]

**S4 Table.** **Differences in homopolymers^a^ found when comparing the UZ1974 isolate to the UW074B genome sequence.**

| Position^b^ | ORF (Gene) | UW074B isolate | UZ1974 isolate |
| --- | --- | --- | --- |
| **49359** | IGR^c^ | 11G | 10G |
| **72679** | IGR^c^ | 9G | 10G |
| **140949** | IGR^c^ | 9C | 10C |
| **156883** | TPASS_20135 | 9G | 10G |
| **208627** | IGR^c^ | 10G | 9G |
| **409141** | IGR^c^ | 10C | 12C |
| **671319** | TPASS_20618 | 9C | 10C |
| **676714** | IGR^c^ | 10C | 9C |
| **937884** | TPASS_20859 | 9G | 10G |
| 1055490 | IGR^c^ | 8C | 10C |

^a^DNA regions containing continuously seven and more identical DNA bases were considered as homopolymeric tracts.

^b^Positions in bold were also found as differences in homopolymers in other study [19].

^c^IGR – Intergenic region.
